# Transtheoretical model to predict the stages of changes in smoking cessation behavior among adolescents

**DOI:** 10.3389/fpubh.2024.1399478

**Published:** 2024-07-16

**Authors:** Min Hee Park, Hye-Young Song

**Affiliations:** ^1^Department of Nursing, Wonkwang University, Iksan, Republic of Korea; ^2^College of Nursing, Woosuk University, Jeonju, Republic of Korea

**Keywords:** adolescents, stages of changes in smoking cessation behaviors, transtheoretical model, smoking, South Korea

## Abstract

**Introduction:**

Previous studies on adolescent smoking cessation behavior based on the transtheoretical model have primarily focused on the development of cessation programs. Attempting to quit smoking is a prerequisite for executing smoking cessation. Appropriate methods must be selected based on the characteristics and intentions of individuals to increase smokers’ satisfaction and success rates in quitting. Therefore, this study aimed to identify changing processes influencing the stages of change associated with successful smoking cessation among adolescents and examined the related factors. This descriptive study explored a transtheoretical model of different stages of changes in smoking cessation behavior among adolescent smokers.

**Methods:**

The participants included 237 middle and high school students in South Korea. We examined the differences in stages of changes in smoking cessation behaviors based on general characteristics, smoking-related characteristics, smoking cessation change processes (cognitive and behavioral), smoking decisional balance (pros and cons of smoking), and self-efficacy.

**Results:**

The probability of reaching the preparation stage of smoking cessation was significantly lower among participants who did not know their father’s educational level than among those who knew their father’s educational level. Conversely, this probability was significantly higher among participants whose mothers had a college or higher education level and those who did not know their mother’s educational level than in those whose mothers had a high school or lower education level.

**Conclusion:**

These findings indicated that parental education is a significant predictor of the preparation stage of smoking cessation, highlighting the importance of the family environment in facilitating smoking cessation among adolescents. Accordingly, communication and support at home could help reinforce adolescents’ determination to quit smoking and make relevant plans. Thus, smoking cessation support should stress the need to enhance communication at home.

## Introduction

The smoking behavior of adolescents is influenced by a complex interplay of psychological, social, and environmental factors. From a developmental perspective, adolescents undergo a higher proportion of substantial physical, psychological, and social changes than any other age group. Adolescence is characterized by an expansion of social networks through interactions with family, friends, schools, and neighborhoods (1). Diverse changes experienced during adolescence include the pursuit of emotional and behavioral independence from parental protection received during childhood, the establishment of autonomy and identity, and the acquisition of the ability to form intimate interpersonal relationships (2, 3).

These developmental changes and numerous societal demands act as stressors for adolescents (4). Owing to environmental characteristics and cognitive immaturity, adolescents possess limited capacity to cope with stress and are at a high risk of expressing emotions in an impulsive and maladaptive manner (5). Smoking is recognized as one consequence of failing to appropriately manage a wide array of emotions during adolescence and responding in inappropriate ways (4, 6).

In South Korea in 2022, the smoking rates among middle and high school students were 6.2% for males and 2.7% for females, showing a decrease from 7.0 and 3.3%, respectively (7). However, the declining age of smoking initiation is 13.5 years old (8, 9). Smoking initiated during adolescence is difficult to quit, leading to a lifetime of smoking. Furthermore, the incidence of smoking-related physical morbidities increases with younger age at smoking initiation, thus increasing the future disease burden (9, 10). In addition, adolescent smoking is associated with issues such as alcohol consumption, nicotine addiction, anxiety, diminished academic performance, and school violence (5), emphasizing the importance of smoking cessation during adolescence.

According to the Korean Youth Risk Behavior Survey, the rate of attempted smoking cessation among adolescent smokers is relatively high at 59.8% (7). However, the success rate of quitting smoking remains low at approximately 10%, given the strong influence of peers and lack of motivation to quit (8). This study highlights the challenges adolescents face in quitting smoking.

Various factors are associated with adolescents’ attempts to quit smoking (11–15). A systematic review analyzing predictors of adolescent smoking cessation found that smoking frequency, having friends and family members who smoke, smoking cessation education in schools, and parental smoking cessation education were associated with attempts to quit smoking (12). Park (13) reported daily smoking quantity, smoking among close friends and family, exposure to smoking cessation education at school, and exposure to anti-smoking media as associated factors. Yim and Park (15) identified the timing of smoking initiation, smoking among friends, vigorous physical activity, experiences of depression, and smoking cessation education exposure as related factors. Moreover, Park et al. (14) found that grade level, subjective academic performance, perceived stress, ease of buying cigarettes, and awareness of cigarette pack warnings were factors associated with the attempts to quit smoking. Thus, attempts to quit smoking among adolescents are associated with diverse factors, including individual smoking-related characteristics, psychological traits, and social attributes.

Prochaska and DiClemente (16) developed a transtheoretical model that emphasizes the need for smoking cessation programs tailored to an individual’s stage of smoking behavior and intention to quit. This model suggests that smoking cessation is not a dichotomous outcome of success or failure, but is a process that includes various stages of change in an attempt to quit smoking (16, 17).

The transtheoretical model outlines a series of stages—precontemplation, contemplation, preparation, action, and maintenance—through which change unfolds (16, 17). The model introduces concepts of stages of change, decisional balance, self-efficacy, and processes of change. Adolescents engage in more frequent attempts to quit smoking as they proceed from the precontemplation stage to the maintenance stage (18). Within this transtheoretical process of change, enhancing self-efficacy can boost motivation to quit smoking. The decisional balance allows adolescents to weigh the losses from smoking against the gains from quitting, thereby facilitating their smoking cessation (19).

Prior studies grounded in the transtheoretical model have primarily focused on the development of smoking cessation programs (19–21). Lee and Song (20) reported that a smoking cessation program based on the transtheoretical model was effective in modifying smoking behavior and socio-psychological variables among high school students. Chae and Choe (19) constructed a smoking cessation class rooted in the model and implemented it among vocational high school students. The authors reported that the program led to positive shifts in the stages of change regarding smoking behavior, enhanced self-efficacy, and reduced smoking behavior. Oh (21) developed and administered a smoking cessation program incorporating the transtheoretical model and physical exercise for female high school students, identifying positive outcomes, such as reduced smoking behavior, depression, and stress; improved self-efficacy; and no significant change in body mass index.

Successful attempts to quit smoking are a prerequisite to quitting. Selecting the appropriate approach tailored to the smoker’s characteristics and intent to quit is crucial for enhancing the satisfaction and success rates of quitting (11, 22). Therefore, in the current study, we aimed to identify the processes of change and associated factors that influence the stages leading to successful cessation among adolescents. We seek to categorize adolescent smoking cessation behaviors according to their stages of change by implementing the transtheoretical model as a framework and identifying the factors that predict each stage of change in smoking behavior.

The current study examined the factors of the transtheoretical model that predict the stages of change in smoking cessation behaviors among adolescent smokers. Specifically, we aimed to identify (1) differences in general characteristics and smoking traits based on the stages of change in smoking cessation behavior; (2) differences in the processes of change, decisional balance, and self-efficacy among adolescents across the stages of changes in smoking cessation behavior; and (3) variables of the transtheoretical model that impact each stage of change in smoking cessation behavior.

## Methods

### Study design

The current study was a descriptive survey.

### Participants

Adolescent smokers registered with a smoking cessation center in 17 metropolitan cities in Korea. Data were collected from 316 participants using a self-report questionnaire. The sample size was determined using the G*Power 3.1 software. Based on Cohen’s grounds for sample size determination, the minimum sample size for a logistic regression with a medium effect size, odds ratio (OR) of 1.6, significance level of 0.05, power of 0.80, and two-tailed test (23) was calculated to be 223 (24). After excluding individuals who were not high school students and those who had not smoked conventional cigarettes, liquid e-cigarettes, or heated tobacco products in the past 30 days, 237 participants were included in the study. The participants were divided according to the stages of changes in the transtheoretical model (16, 17). The participants comprised 77 individuals in the preparation stage, 56 in the contemplation stage, 72 in the precontemplation stage, and 32 with no plans to quit.

### Instruments

#### Stages of changes in smoking cessation behavior

Participants who stated their plans to quit smoking within one and six months were likely in the preparation and contemplation stages, respectively. Participants who intended to quit in the future were categorized as being in the precontemplation stage. Finally, participants who had no plans to quit smoking were categorized as having no plans to quit.

### General characteristics

General characteristics included age, sex (men/women), educational level (middle school/high school), economic level (medium or high/low), academic performance (medium or high/low), living arrangements (living with parents/not living with parents), father’s educational level (high school or lower/college or higher/unknown), mother’s educational level (high school or lower/college or higher/unknown), self-rated health (SRH), current drinking status, vigorous physical activity, breakfast consumption, and perceived stress.

The following question assessed economic level: “What was your family’s economic status in the last 12 months?” and the responses were categorized as “high,” “medium,” or “low.” The academic performance was assessed “How did you do in school in the last 12 months?” with responses of “high,” “medium,” and “low.” SRH was assessed “How would you rate your health?” The responses were categorized into “healthy,” “average,” and “unhealthy.” Drinking was assessed using the following question: “How many days have you had at least one alcoholic drink in the past 30 days?” A subject with no alcohol consumption in the past 30 days was defined as a “non-drinker,” whereas a subject who consumed at least one drink or more was defined as a “drinker.” Vigorous physical activity was assessed using the following question: “How many days in the past seven days have you performed vigorous physical activity that made you extremely short of breath or sweat?” Three days or more was defined as “yes,” whereas fewer than three days was defined as “no.” Breakfast consumption was assessed using the following question: “How many days have you had breakfast (excluding days when you only had milk or juice) in the past seven days?” Responses of 0–4 days were defined as “no,” whereas five days or more were defined as “yes.” Perceived stress was assessed using the following question: “How stressed are you on a normal day?” Responses indicating “extremely,” “a lot,” and “a little” were categorized as “yes,” whereas responses indicating “not much” and “not at all” were categorized as “no.”

### Smoking-related characteristics

Smoking-related characteristics included age at smoking initiation, family’s smoking, friends’ smoking, prior smoking cessation education, and awareness of smoking cessation ads.

Age at smoking initiation was determined using the following questions: “When did you first try a conventional cigarette (even just a puff or two)?”; “When did you first use a liquid e-cigarette?”; and “When did you first use a heated tobacco product?” Regarding family’s smoking, participants indicated if any family members currently smoke, with responses categorized as “no” if none were indicated and “yes” if at least one family member was reported as a smoker. Friends’ smoking was assessed by asking, “Do any of your close friends smoke?” Responses were divided into “most do not smoke” and “most smoke.” Prior smoking cessation education was classified based on whether the participant had received any smoking prevention or cessation education at school in the past 12 months. Awareness of smoking cessation ads was categorized based on whether the participant had seen or heard any smoking cessation-related ads in the past 12 months.

### Variables of the transtheoretical model

The variables of the transtheoretical model used in the current study were processes of changes in smoking cessation (including cognitive and behavioral changes), decisional balance for smoking (pros and cons of smoking), and self-efficacy.

The processes of changes in smoking cessation refer to the adaptive mechanisms used to alter one’s smoking behavior toward cessation. The simplified tool developed by Prochaska and DiClemente (16) was used to measure the process of cognitive (10 items) and behavioral (10 items) changes. Each item indicating action or experience related to smoking in the past month was rated on a five-point Likert scale (1 = not at all; 5 = very frequently), with higher scores indicating more frequent application of that change process. The total score was 10–50 and 10–50 for cognitive and behavioral change processes, respectively. The Cronbach’s alpha was 0.86 at the time of development and 0.89 and 0.88 for cognitive and behavioral changes, respectively, in this study.

Decisional balance for smoking is a variable determining smoking-related decisions and consists of perceived pros and cons of smoking. It was assessed using the smoking decisional balance scale (SDB) (25), comprising 10 items for the pros and 10 items for the cons of smoking. Items were rated on a five-point Likert scale (1 = not important at all; 5 = very important). Higher scores indicated perceiving more pros or cons of smoking. The Cronbach’s alpha was 0.87 for the pros of smoking and 0.90 for the cons of smoking at the time of development, with a value of 0.90 in the current study.

Self-efficacy is one’s ability to refrain from smoking in specific situations, assessed using the nine-item smoking cessation self-efficacy measure (26). Items were rated on a five-point Likert scale (1 = not confident at all; 5 = very confident). The scores were 5–45, with higher scores indicating greater self-efficacy. The Cronbach’s alpha was 0.98 at the time of development, with a value of 0.93 in the current study.

### Data collection and ethical considerations

Data were collected through an online survey from September 2022 to February 2023 through 17 smoking cessation centers nationwide. The Institutional Review Board (IRB) of Wonkwang University approved the study before data collection (WKIRB-202208-SB-068). The purpose and content of this study were communicated to the representatives of the 17 smoking cessation centers via official letters and emails, followed by a telephone explanation of the data collection procedure. Data were collected only if both guardians and adolescents voluntarily agreed to participate in the study. The online study information sheet provided an explanation of the study’s purpose and content, assurance that participants would not be subject to any harm, and confidentiality. The participants were also informed of their freedom to withdraw at any time and were not obliged to answer any questions they preferred not to. All participants provided informed consent online before participation. No personally identifiable information, such as names or phone numbers, was collected. Collected data were anonymized and coded for computer processing, with plans to dispose of the data after three years. After completing the survey, participants were provided an online gift coupon as a token of appreciation.

### Data analysis

Data were analyzed using the SPSS Statistics 26.0 SPSS (IBM Corporation, Armonk, New York, USA), and significance was established at 0.05. The participants’ general characteristics and differences in the stages of changes in smoking cessation behaviors based on the general characteristics were analyzed using a chi-square test. Differences in stages of changes in smoking cessation behaviors based on age, age at smoking initiation, processes of change, decisional balance, and self-efficacy were analyzed using a one-way analysis of variance (ANOVA). Significant variables were further analyzed using the Scheffé post-hoc test. Finally, the predictors of each stage of change in smoking cessation behavior were analyzed using multinomial logistic regression analysis with the preceding stage used as reference variables.

## Results

### Stages of changes in smoking cessation behavior based on general and smoking-related characteristics

The general and smoking-related characteristics of the study subjects are as follows.

The average age of the participants in this study was 17.49 years old, with 59.9% male and 40.1% female. 19.0% were middle school students and 81.0% were high school students. Academic performance were most likely to be “low” at 52.7%, and economic status was most likely to be “high” or “middle” at 85.7%. 78.9% of subjects lived with their parents, with 37.1% of fathers having a high school diploma or less and 39.2% of mothers having a college degree or higher. Subjectively, 46.0% of the subjects reported being “healthy,” 61.6% of the subjects drank alcohol, only 24.5% of the subjects reported engaging in vigorous physical activity, 75.1% of the subjects reported not eating breakfast, and 40.9% of the subjects reported experiencing stress. The average age of smoking initiation was 14.13 years, 63.7% of subjects had a family member who smoked, and 68.8% of subjects reported that most of their friends smoked. In the past 12 months, 72.2% of participants had received smoking cessation education, and 65% had seen or heard a smoking cessation advertisement in the past 12 months.

The stages of changes in smoking cessation behavior based on academic performance (χ^2^ = 10.90, *p* = 0.012) were as follows. In the no plans to quit, precontemplation, contemplation, and preparation groups had 25.0, 43.1, 50.0, and 58.4% participants, respectively, who exhibited average or higher performance. These results suggested that academic performance generally improves toward the preparation stage (Table 1). While none of the variables showed statistically significant differences, the preparation group had the highest percentage of healthy people (55.8%) and the highest percentage of non-drinkers (46.2%).

**Table 1:** General characteristics and smoking-related characteristics according to stages of changes in smoking cessation behavior.

Variable	Category	Total	Stages of changes in smoking cessation behavior				χ^2^/*F* (*p*)
			Preparation (*n* = 77, 32.5%)	Contemplation (*n* = 56, 23.6%)	Precontemplation (*n* = 72, 30.4%)	No plans (*n* = 32, 13.5%)	
General characteristics							
Age		17.49 ± 1.60	17.40 ± 2.22	17.70 ± 1.19	17.36 ± 1.27	17.66 ± 1.07	0.65 (0.583)
Sex	Men	142 (59.9)	54 (70.1)	34 (60.7)	35 (48.6)	19 (59.4)	7.20 (0.066)
	Women	95 (40.1)	23 (29.9)	22 (39.3)	37 (51.4)	13 (40.6)	
Educational level	Middle school	45 (19.0)	12 (15.6)	10 (17.9)	19 (26.4)	4 (12.5)	4.07 (0.254)
	High school	192 (81.0)	65 (84.4)	46 (82.1)	53 (73.6)	28 (87.5)	
Academic performance	Medium or high	112 (47.3)	45 (58.4)	28 (50.0)	31 (43.1)	8 (25.0)	10.90 (0.012)
	Low	125 (52.7)	32 (41.6)	28 (50.0)	41 (56.9)	24 (75.0)	
Economic level	Medium or high	203 (85.7)	65 (84.4)	51 (91.1)	59 (81.9)	28 (87.5)	2.33 (0.507)
	Low	34 (14.3)	12 (15.6)	5 (8.9)	13 (18.1)	4 (12.5)	
Living arrangement	Live with parents	187 (78.9)	62 (80.5)	46 (82.1)	56 (77.8)	23 (71.9)	1.48 (0.687)
	Not live with parents	50 (21.1)	15 (19.5)	10 (17.9)	16 (22.2)	9 (28.1)	
Father’s education	High school or lower	88 (37.1)	29 (37.7)	23 (41.1)	26 (36.1)	10 (31.3)	5.48 (0.484)
	College or higher	87 (36.7)	28 (36.4)	24 (42.9)	25 (34.7)	10 (31.3)	
	Do not know	62 (26.2)	20 (26.0)	9 (16.1)	21 (29.2)	12 (37.5)	
Mother’s education	High school or lower	81 (34.2)	30 (39.0)	20 (35.7)	22 (30.6)	9 (28.1)	3.27 (0.775)
	College or higher	93 (39.2)	31 (40.3)	19 (33.9)	29 (40.3)	14 (43.8)	
	Do not know	63 (26.6)	16 (20.8)	17 (30.4)	21 (29.2)	9 (28.1)	
SRH	Healthy	109 (46.0)	43 (55.8)	28 (50.0)	23 (31.9)	15 (46.9)	11.18 (0.083)
	Average	93 (39.2)	23 (29.9)	23 (41.1)	36 (50.0)	11 (34.4)	
	Unhealthy	35 (14.8)	11 (14.3)	5 (8.9)	13 (18.1)	6 (18.8)	
Drinking status	Non-drinker	91 (38.4)	36 (46.8)	20 (35.7)	24 (33.3)	11 (34.4)	3.44 (0.328)
	Drinker	146 (61.6)	41 (53.2)	36 (64.3)	48 (66.7)	21 (65.6)	
Vigorous physical activity	No	179 (75.5)	55 (71.4)	45 (80.4)	56 (77.8)	23 (71.9)	1.84 (0.607)
	Yes	58 (24.5)	22 (28.6)	11 (19.6)	16 (22.2)	9 (28.1)	
Eating breakfast	No	178 (75.1)	59 (76.6)	40 (71.4)	55 (76.4)	24 (75.0)	0.56 (0.905)
	Yes	59 (24.9)	18 (23.4)	16 (28.6)	17 (23.6)	8 (25.0)	
Stress	Yes	97 (40.9)	32 (41.6)	23 (41.1)	30 (41.7)	12 (37.5)	0.19 (0.980)
	No	140 (59.1)	45 (58.4)	33 (58.9)	42 (58.3)	20 (62.5)	
Smoking-related characteristics							
Age at smoking initiation		14.13 ± 1.70	14.30 ± 1.86	14.36 ± 1.81	13.93 ± 1.39	13.78 ± 1.74	1.34 (0.254)
Family’s smoking	No	66 (27.8)	28 (36.4)	17 (30.4)	17 (23.6)	4 (12.5)	9.23 (0.161)
	Yes	151 (63.7)	43 (55.8)	36 (64.3)	49 (68.1)	23 (71.9)	
	Do not know	20 (8.4)	6 (7.8)	3 (5.4)	6 (8.3)	5 (15.6)	
Friends’ smoking	Most do not smoke	74 (31.2)	25 (32.5)	17 (30.4)	22 (30.6)	10 (31.3)	0.09 (0.993)
	Most smoke	163 (68.8)	52 (67.5)	39 (69.6)	50 (69.4)	22 (68.8)	
Prior smoking cessation education	No	66 (27.8)	17 (22.1)	17 (30.4)	21 (29.2)	11 (34.4)	2.19 (0.533)
	Yes	171 (72.2)	60 (77.9)	39 (69.6)	51 (70.8)	21 (65.6)	
Awareness of smoking cessation promotions	No	83 (35.0)	30 (39.0)	12 (21.4)	26 (36.1)	15 (46.9)	7.09 (0.069)
	Yes	154 (65.0)	47 (61.0)	44 (78.6)	46 (63.9)	17 (53.1)	

### Stages of changes in smoking cessation behavior based on process of change, decisional balance, and self-efficacy

The results identified stages of changes in smoking cessation behavior according to cognitive change process (*F* = 11.00, *p* < 0.001), behavioral change process (*F* = 13.50, *p* < 0.001), pros of smoking (*F* = 4.35, *p* = 0.005), and self-efficacy (*F* = 8.32, *p* < 0.001). According to the Scheffé post-hoc comparison, the cognitive change process was significantly higher in the precontemplation, contemplation, and preparation stages than in the no plans to quit stage and in the preparation stage than in the precontemplation stage. The behavioral change process was significantly higher in the contemplation and preparation stages than in the no plans to quit stage and in the preparation stage than in the precontemplation stage. Perceived pros of smoking were significantly higher in the precontemplation and no plans to quit stage than in the preparation stage. Self-efficacy was significantly higher in the preparation stage than in the no plans to quit or precontemplation stages (Table 2).

**Table 2:** Change process, decisional balance, and self-efficacy according to stages of changes in smoking cessation behavior.

Variable	Total	Stages of changes in smoking cessation behavior				*F* (*p*) Scheffé
		Preparation (a)	Contemplation (b)	Precontemplation (c)	No plans (d)	
Cognitive change process	2.87 ± 0.73	3.14 ± 0.70	2.95 ± 0.58	2.75 ± 0.77	2.35 ± 0.63	11.00 (<0.001) d < c,b,a/c < a
Behavioral change process	2.78 ± 0.72	3.10 ± 0.68	2.89 ± 0.59	2.55 ± 0.72	2.35 ± 0.68	13.50 (<0.001) d < b,a/c < a
Pros of smoking	2.98 ± 0.68	2.80 ± 0.76	2.93 ± 0.43	3.15 ± 0.70	3.15 ± 0.66	4.35 (0.005) a < c,d
Cons of smoking	3.21 ± 0.65	3.24 ± 0.73	3.20 ± 0.46	3.30 ± 0.68	2.99 ± 0.62	1.73 (0.162)
Self-efficacy	2.79 ± 0.95	3.16 ± 0.85	2.85 ± 0.79	2.52 ± 0.96	2.41 ± 1.12	8.32 (<0.001) d,c < a

### Predictors of stages of changes in smoking cessation behavior

Multinomial logistic regression was performed to identify the predictors of the stages of changes in smoking cessation behavior. With the stages of changes in smoking cessation behavior set as the dependent variable, we calculated ORs for the precontemplation stage relative to having no plan to quit, the contemplation stage relative to the precontemplation stage, and the preparation stage relative to the contemplation stage. As age and educational level were similar variables, we only used the educational level to prevent multicollinearity.

First, the predictors of the precontemplation stage were analyzed. The probability of reaching the precontemplation stage was significantly lower with higher perceived pros of smoking (OR = 0.20, *p* = 0.048), whereas the probability of reaching the precontemplation stage was significantly higher with higher perceived cons of smoking (OR = 8.36, *p* = 0.019). The probability of reaching the precontemplation stage was significantly higher among participants with good academic performance (OR = 4.89, *p* = 0.035).

Next, the predictors of the contemplation stage were analyzed. The probability of reaching the contemplation stage was significantly lower with higher perceived pros of smoking (OR = 0.27, *p* = 0.021), with not knowing the father’s educational level (OR = 0.18, *p* = 0.040), and with vigorous physical activity (OR = 0.23, *p* = 0.045).

Finally, the predictors of the preparation stage were analyzed. The probability of reaching the preparation stage was significantly lower with not knowing the father’s educational level (OR = 0.17, *p* = 0.032); contrastingly, the probability was significantly higher with mothers having college or higher education (OR = 4.21, *p* = 0.044) or not knowing the mother’s educational level (OR = 8.54, *p* = 0.009) compared with mothers’ having a high school education or lower (Table 3).

**Table 3:** Predictors of the stages of changes in smoking cessation behavior stages.

Variable	Precontemplation		Contemplation		Preparation	
	OR (95% CI)	*p*	OR (95% CI)	*p*	OR (95% CI)	*p*
Cognitive change process	7.45 (0.81–68.61)	0.076	0.79 (0.23–2.69)	0.704	1.26 (0.28–5.64)	0.765
Behavioral change process	0.23 (0.03–1.84)	0.166	3.07 (0.90–10.48)	0.073	1.06 (0.24–4.74)	0.937
Pros of smoking	0.20 (0.04–0.99)	0.048	0.27 (0.09–0.82)	0.021	0.59 (0.25–1.35)	0.210
Cons of smoking	8.36 (1.42–49.22)	0.019	1.32 (0.41–4.22)	0.640	0.91 (0.37–2.26)	0.841
Self-efficacy	0.92 (0.45–1.89)	0.823	1.55 (0.85–2.80)	0.152	1.90 (0.96–3.78)	0.067
Gender (man)	0.59 (0.15–2.41)	0.467	0.97 (0.37–2.55)	0.953	2.05 (0.76–5.50)	0.153
Economic status (high)	1.98 (0.30–13.09)	0.480	4.44 (0.97–20.43)	0.055	0.33 (0.07–1.50)	0.150
Academic performance (Good)	4.89 (1.12–21.35)	0.035	2.01 (0.74–5.48)	0.174	2.12 (0.83–5.45)	0.118
Living with parents (yes)	1.56 (0.35–7.05)	0.561	0.62 (0.17–2.26)	0.473	1.19 (0.32–4.44)	0.794
Educational level (high school)	0.19 (0.03–1.01)	0.052	1.31 (0.40–4.32)	0.658	0.62 (0.16–2.45)	0.496
Father’s education (≥ college)	3.37 (0.50–22.60)	0.211	0.76 (0.24–2.43)	0.649	0.32 (0.07–1.37)	0.124
Father’s education (unknown)	0.87 (0.17–4.51)	0.867	0.18 (0.04–0.92)	0.040	0.17 (0.04–0.86)	0.032
Mother’s education (≥ college)	0.28 (0.04–1.79)	0.179	0.56 (0.17–1.83)	0.334	4.21 (1.04–16.97)	0.044
Mother’s education (unknown)	1.13 (0.17–7.38)	0.897	2.97 (0.62–14.11)	0.171	8.54 (1.72–42.39)	0.009
SRH (healthy)	0.49 (0.07–3.23)	0.457	2.67 (0.55–12.91)	0.223	0.23 (0.04–1.26)	0.091
SRH (average)	1.07 (0.20–5.78)	0.938	1.48 (0.32–6.81)	0.617	0.22 (0.04–1.21)	0.082
Drinking (yes)	2.06 (0.49–8.71)	0.327	0.83 (0.29–2.34)	0.723	1.68 (0.66–4.26)	0.277
Vigorous physical activity (yes)	0.81 (0.18–3.60)	0.786	0.23 (0.06–0.97)	0.045	0.70 (0.23–2.16)	0.532
Eating breakfast (yes)	0.90 (0.19–4.27)	0.893	1.76 (0.58–5.33)	0.319	2.11 (0.74–6.03)	0.163
Perceived stress (yes)	0.96 (0.29–3.23)	0.949	1.40 (0.53–3.67)	0.494	1.01 (0.39–2.61)	0.991
Age at smoking initiation	1.29 (0.85–1.93)	0.228	1.12 (0.83–1.49)	0.462	0.87 (0.69–1.11)	0.268
Family’s smoking (yes)	0.40 (0.08–1.88)	0.244	0.90 (0.32–2.53)	0.838	0.30 (0.05–1.88)	0.200
Family’s smoking (unknown)	0.37 (0.04–3.40)	0.378	0.50 (0.08–3.16)	0.460	0.19 (0.03–1.13)	0.068
Friends’ smoking (most)	1.23 (0.34–4.50)	0.754	1.23 (0.43–3.48)	0.702	1.02 (0.37–2.81)	0.968
Smoking cessation education (yes)	1.32 (0.36–4.87)	0.677	0.86 (0.32–2.33)	0.773	0.33 (0.11–1.03)	0.057
Smoking cessation ads (yes)	1.72 (0.48–6.24)	0.408	2.22 (0.74–6.62)	0.152	2.36 (0.82–6.85)	0.113

References for categorical variables: stages of changes, no plans to quit; gender, woman; economic status, low; academic performance, below average; living with parents, no; educational level, middle school; parental education, high school or lower; SRH, unhealthy; drinking, non-drinker; vigorous physical activity, no; eating breakfast, no; stress, no; family’s smoking, no; friends’ smoking, most do not smoke; smoking cessation education, no; smoking cessation ads, no.

## Discussion

In the current study, we aimed to categorize adolescents’ smoking cessation behaviors by different stages of change in the transtheoretical model and determine the predictors of each stage of change to gain a clear understanding of smoking cessation behaviors among adolescents.

In the behavioral step-by-step model of the pan-theoretical model, there were many adolescents who practiced smoking cessation behavior in the pre-planning and preparation stages. It is the time to recognize the seriousness of the problem and start to find a solution as the stage of thinking and thinking about a specific action before taking a specific action in the pre-planning stage. This is the process of adolescents’ decisions moving into action.

In the preparation stage for quitting smoking, the individual is actually preparing for a specific action, indicating a state in which the action is likely to take place. Adolescents’ pre-planning and preparation stages are initial preparations before performing a specific action. These steps are the only steps to enter the action stage, contributing to the explanation of the important process of individual behavior change.

Among the general and smoking-related characteristics, stages of changes in smoking cessation behavior significantly differed according to academic performance. Academic performance tended to be better as adolescents progressed from the pre-contemplation stage to the preparation stage of smoking cessation. This suggests that being motivated or setting a goal for smoking cessation and reaching the preparation stage may positively impact academic performance (27). Generally, students who perform well academically demonstrate strong self-control and willpower (15, 16). Therefore, it can be inferred that students with good academic performance are more likely to quit smoking. A good academic performance in the preparation stage for smoking cessation indicates high effort and motivation to attempt quitting smoking (28). Moreover, smoking cessation behavior is associated with cognitive efforts to adopt healthy behaviors (29). Considering the relationship between smoking cessation behavior and academic performance, smoking cessation campaigns and educational programs in schools or communities may positively impact academic outcomes.

Herein, we identified significant differences in the changing cognitive processes, behavioral processes of change, perceived pros of smoking, and self-efficacy across the stages of changes in smoking cessation behavior. Higher levels of cognitive processes of change, behavioral processes of change, perceived benefits of smoking, and self-efficacy were associated more strongly with the preparation stage than the no plan and pre-contemplation stages. The significant differences in cognitive and behavioral change processes indicate that as adolescents progress to the preparation stage of quitting smoking, cognitive and behavioral changes also increase (30). In other words, adolescents establish specific behavioral plans to quit smoking in addition to having the determination to quit smoking.

The significant difference in perceived pros of smoking in the preparation stage suggested that adolescents were aware of the cons or negative smoking aspects and clearly recognized the benefits of quitting (31). Self-efficacy was higher in the preparation stage, suggesting that adolescents’ confidence in quitting smoking was bolstered (32). In other words, adolescents were prepared to quit smoking when they positively evaluated their abilities to quit smoking (33).

These findings indicate that adolescents who make an effort and are motivated to establish smoking cessation plans experience cognitive, behavioral, and psychological changes related to quitting smoking (34). Therefore, programs and support that offer motivation and assist in planning and implementing smoking cessation must be provided for adolescents who are considering quitting smoking.

The probability of reaching the precontemplation stage compared with the no plans to quit stage was significantly lower with perceiving pros of smoking. In other words, establishing plans to quit smoking is difficult when smoking is perceived to have numerous pros (35). Thus, smoking cessation programs for adolescents should stress the detrimental aspects of smoking to lower their perceptions of the pros of smoking. Recognizing the negative smoking aspects would help foster determination to quit smoking (36). The odds of reaching the precontemplation stage of quitting smoking compared with the no plans to quit stage were significantly higher among adolescents who perceive the cons of smoking and those with good academic performance. In other words, students who are more aware of the negative impact of smoking on academic performance tend to consider quitting smoking (37). Accordingly, these students understand that quitting smoking can improve better academic performance (38). The result that the probability of reaching the precontemplation stage is higher with better academic performance suggests that students with positive attitudes toward academics consider quitting smoking (18). Therefore, education and informational campaigns need to inform adolescents regarding the adverse impact of smoking on academic studies and raise awareness that quitting smoking may help enhance academic performance.

Considering the predictors of the contemplation stage compared with the precontemplation stage, the probability of reaching the contemplation stage was significantly lower among adolescents who perceived the pros of smoking, those who did not know their father’s educational level, and those who engaged in vigorous exercise. These results suggest that quitting smoking can be challenging for students who perceive that smoking is associated with numerous pros (39, 40). Thus, the perception of the pros of smoking among those considering to quit need be reduced.

The result that the probability of reaching the contemplation stage of quitting smoking was lower among adolescents who did not know their father’s educational level suggested that family environment influenced the students’ willingness to quit smoking (41). A lack of knowledge regarding the educational level of their father could indicate a lack of communication or support within the home, which, in turn, could negatively affect the motivation to quit smoking (37). The result that engaging in vigorous exercise was associated with a lower likelihood of being in the contemplation stage of quitting smoking aligned with prior findings that an active lifestyle or exercise may negatively impact the formation of quitting plans (42). This could be attributed to the fact that vigorous exercise is used as a method of stress relief, and smoking is also concurrently used as a strategy for managing stress.

Reducing the perceived benefits of smoking, enhancing communication and support within the family environment, and incorporating positive stress management strategies into an active lifestyle should be considered for successfully achieving a smoking cessation plan. Moreover, quitting plans must be supported by strengthening communication within the family and utilizing smoking cessation support programs or resources.

Regarding the predictors of the preparation stage of quitting smoking compared with the contemplation stage, the probability of reaching the preparation stage was significantly lower among adolescents who did not know their father’s educational level. Furthermore, the probability of reaching the preparation stage was significantly higher among adolescents with a mother who had a college or higher educational level and those who did not know their mother’s educational level than those with mothers with a high school or lower educational level. As highlighted earlier, adolescents who do not know their father’s educational level may be unmotivated to quit smoking and make plans to quit owing to a lack of communication or support within their family (39). The results that the likelihood of reaching the preparation stage was higher when the mother had a higher educational level or when her educational level was unknown suggest that higher maternal education may correlate with greater awareness and support for quitting smoking within the home (42, 43). Parental education is also related to socioeconomic factors (38).

Adolescent smoking cessation education plays an important role in establishing social norms, expectations, and social support systems (37, 38). Adolescents are given the opportunity to receive social support when they attempt to quit smoking even if they do not start smoking or have already started, depending on the influence of the social network (35, 41). The government’s support for smoking cessation policy can create a social environment in which young people can be supported. These points show that knowledge education for youth smoking cessation can affect not only their personal choices but also their social environment (39, 40). Therefore, youth smoking cessation requires efforts from families, schools, and communities

## Conclusion and recommendations

The objective of the current study was to identify the factors of the transtheoretical model that predict different stages of changes in smoking cessation behaviors among adolescent smokers. We analyzed adolescents’ general and smoking-related characteristics and transtheoretical model factors that influenced the stages of changes in smoking cessation behavior. The results indicate that parental education substantially predicts the preparation stage of quitting smoking among adolescents. This highlights the importance of adolescents’ family environment in preparation to quit smoking. Family communication and support may play pivotal roles in reinforcing adolescents’ determination to quit smoking and make related plans. Thus, smoking cessation support programs and campaigns should emphasize the need to enhance communication and support for quitting smoking at home.

This study had several limitations. First, our study findings have limited generalizability, as participants were recruited from a subset of regions. Thus, future studies should include participants from more diverse regions to identify the predictors of smoking cessation behaviors among adolescents. Second, the causality among the variables could not be determined owing to the cross-sectional nature of the study. Third, future studies should employ objective rather than self-reported variables for smoking-related characteristics to obtain accurate measurements.

## Data availability statement

The original contributions presented in the study are included in the article/supplementary material, further inquiries can be directed to the corresponding author.

## Ethics statement

The studies involving humans were approved by the Institutional Review Board (IRB) of Wonkwang University before data collection (WKIRB-202208-SB-068). The studies were conducted in accordance with the local legislation and institutional requirements. Written informed consent for participation in this study was provided by the participants’ legal guardians/next of kin. Written informed consent was obtained from the individual(s), and minor(s)’ legal guardian/next of kin, for the publication of any potentially identifiable images or data included in this article.

## Author contributions

MP: Writing – original draft. H-YS: Writing – review & editing.
